# Effects of short-term dry immersion on bone remodeling markers, insulin and adipokines

**DOI:** 10.1371/journal.pone.0182970

**Published:** 2017-08-14

**Authors:** Marie-Thérèse Linossier, Liubov E. Amirova, Mireille Thomas, Myriam Normand, Marie-Pierre Bareille, Guillemette Gauquelin-Koch, Arnaud Beck, Marie-Claude Costes-Salon, Christine Bonneau, Claude Gharib, Marc-Antoine Custaud, Laurence Vico

**Affiliations:** 1 University of Lyon, Medicine Faculty of Saint-Etienne, INSERM, UMR 1059, Saint Etienne, France; 2 University of Angers, Medicine Faculty, Mitovasc Laboratory, UMR CNRS 6015, INSERM 1083, Angers, France; 3 Institute of Biomedical Problems, Russian Academy of Sciences, Moscow, Russia; 4 Institute of Space Physiology and Medicine (MEDES), Toulouse, France; 5 French Space Agency (CNES), Paris, France; 6 University Hospital, Saint-Etienne, France; 7 University of Claude Bernard, Lyon, France; 8 Clinical Research Center, CHU d'Angers, Angers, France; Cinvestav-IPN, MEXICO

## Abstract

**Background:**

Dry immersion (DI), a ground-based model of microgravity previously used in Russia, has been recently implemented in France. The aim of this study was to analyze early events in a short-term DI model in which all conditions are met to investigate who is first challenged from osteo- or adipo-kines and to what extent they are associated to insulin-regulating hormones.

**Methods:**

Twelve healthy men were submitted to a 3-day DI. Fasting blood was collected during pre-immersion phase for the determination of the baseline data collection (BDC), daily during DI (DI_24h_, DI_48H_ and DI_72h_), then after recovery (R_+3h_ and R_+24h_). Markers of bone turnover, phosphocalcic metabolism, adipokines and associated factors were measured.

**Results:**

Bone resorption as assessed by tartrate-resistant acid phosphatase isoform 5b and N-terminal crosslinked telopeptide of type I collagen levels increased as early as DI_24h_. At the same time, total procollagen type I N- and C-terminal propeptides and osteoprotegerin, representing bone formation markers, decreased. Total osteocalcin [OC] was unaffected, but its undercarboxylated form [Glu-OC] increased from DI_24h_ to R_+3h_. The early and progressive increase in bone alkaline phosphatase activities suggested an increased mineralization. Dickkopf-1 and sclerostin, as negative regulators of the Wnt-β catenin pathway, were unaltered. No change was observed either in phosphocalcic homeostasis (calcium and phosphate serum levels, 25-hydroxyvitamin D, fibroblast growth factor 23 [FGF23]) or in inflammatory response. Adiponectemia was unchanged, whereas circulating leptin concentrations increased. Neutrophil gelatinase-associated lipocalin [lipocalin-2], a potential regulator of bone homeostasis, was found elevated by 16% at R_+3h_ compared to DI_24h_. The secretory form of nicotinamide phosphoribosyl-transferase [visfatin] concentrations almost doubled after one day of DI and remained elevated. Serum insulin-like growth factor 1 levels progressively increased. Fasting insulin concentrations increased during the entire DI, whereas fasting glucose levels tended to be higher only at DI_24h_ and then returned to BDC values. Changes in bone resorption parameters negatively correlated with changes in bone formation parameters. Percent changes of ultra-sensitive C-reactive protein positively correlated with changes in osteopontin, lipocalin-2 and fasting glucose. Furthermore, a positive correlation was found between changes in FGF23 and Glu-OC, the two main osteoblast-/osteocyte-derived hormones.

**Conclusion:**

Our results demonstrated that DI induced an unbalanced remodeling activity and the onset of insulin resistance. This metabolic adaptation was concomitant with higher levels of Glu-OC. This finding confirms the role of bone as an endocrine organ in humans. Furthermore, visfatin for which a great responsiveness was observed could represent an early and sensitive marker of unloading in humans.

## Introduction

Exposure to microgravity induces modification of physiological systems. Experimental opportunities during actual space flight are limited, so ground-based models are necessary. The most used system for more than 20 years to expose the skeleton to reduced gravity stimulation has been the head-down tilt bed rest. Unlike bed rest, the dry immersion (DI) model developed by Russian scientists during the early 1970s but less well known elsewhere provides a unique opportunity to study the physiological effects of the lack of a supporting structure for the body. The absence of mechanical support of specific zones during immersion creates a state akin to weightlessness called “supportlessness”. Therefore, the effects of DI are expected to be more severe and/or earlier than in support-loaded bed rest.

Bone and calcium metabolism during bed rest studies has been well investigated in volunteers. Calcium excretion is increased from the beginning of bed rest leading to a sustained negative calcium balance. Calcium absorption is also reduced. After bed rest lasting for several weeks, bone density, stiffness of bones of the lower limbs and lumbar vertebrae, and bone architecture are altered [1,2]. A pronounced increase in bone resorption markers already occurs after 24h of immobilization and induces a significant increase in osteoclastic activity [3,4]. At the same time, bone formation markers remain unchanged or decreased [5]. Accompanying these alterations, it is consistently reported that body weight, muscle mass and muscle strength are reduced. Furthermore, the development of insulin resistance was observed as early as after 3 days of bed rest [6].

The relationship between bone and energy metabolism has now been recognized. However, it has been poorly studied during physical inactivity induced by microgravity-simulated conditions. Regulation of energy metabolism has recently expanded to include the involvement of bone in regulating glucose homeostasis, both as a target for insulin and as an endocrine organ. Osteocalcin (OC), which is stored in the bone matrix, is released by bone resorption in its undercarboxyalated form (the active form) and targets GPRC6A receptors on pancreatic β cells to increase insulin secretion and on muscle and white adipose tissue to promote glucose homeostasis. OC action on the pancreas causes an increase in insulin secretion and in sensitivity and β-cell proliferation as shown by experiments using OC^-/-^ mice [7,8]. Thus, total and decarboxylated OC along with adipokines with potential roles on bone cellular activities definitively need to be evaluated in microgravity-simulated situations in which resorption activity has been shown to be greatly increased. Another hormone produced by osteocytes is fibroblast growth factor 23 (FGF23). FGF23 is best known for its role in various disorders of mineral metabolism [9]. However, recent studies have found an association between circulating levels of FGF23, parathyroid hormone (PTH), leptin and insulin sensitivity [10,11]. It has been recently shown that a strong metabolic effort of 21 days of cycling race was related to an increase in FGF23 in plasma [12]. No data regarding inactivity in human have been published to our knowledge. Thus, in microgravity-simulated models, all conditions are met to analyze who is first challenged from osteo- or adipo-kines and to what extent they are associated to insulin-regulating hormones.

Thus, the aim of this study was to analyze early events in bone cellular activities in relation to adipokines and their regulators in order to clarify their potential associations in unloading-induced altered energy metabolism. Doing so at the initiation of unloading is of great importance to better understand potential causative factors and consequences in a longer trial. Short-term DI represents the model adapted to such an approach to better understand consequences of severe unloading.

## Material and methods

The DI study took place at the Institute for Space Medicine Physiology, MEDES Clinique d’investigation in Toulouse, France. This study was conducted under the leadership of the French and European Space Agencies. Study design was established in accordance with the Declaration of Helsinki guidelines for research on human subjects (1989) and was approved by the National Agency for the Safety of Medicines and Health Products (ANSM) as well as the local ethics committee (CPP Sud Ouest Outre-Mer I, France). All the subjects were asked to provide informed written consent before participating in this study. The written consent forms are stored at the MEDES clinic in Toulouse.

### Subjects

Medically and psychologically healthy male volunteers (n = 12) were recruited to undergo 3 days of DI (age 31.8±1.4 years, body height 178±2 cm, body weight 75±2 kg and body mass index 23.6±0.4 kg.m^-2^, VO_2max_ 38.8±1.1 ml.min^-1^.kg^-1^, mean± SEM). All participants were non smokers, taking no medication or drugs. None of the men had any disorders that affected their metabolism of calcium or bone, or any history of endocrine, renal or liver illness.

### General description of the study

**Fig 1:**
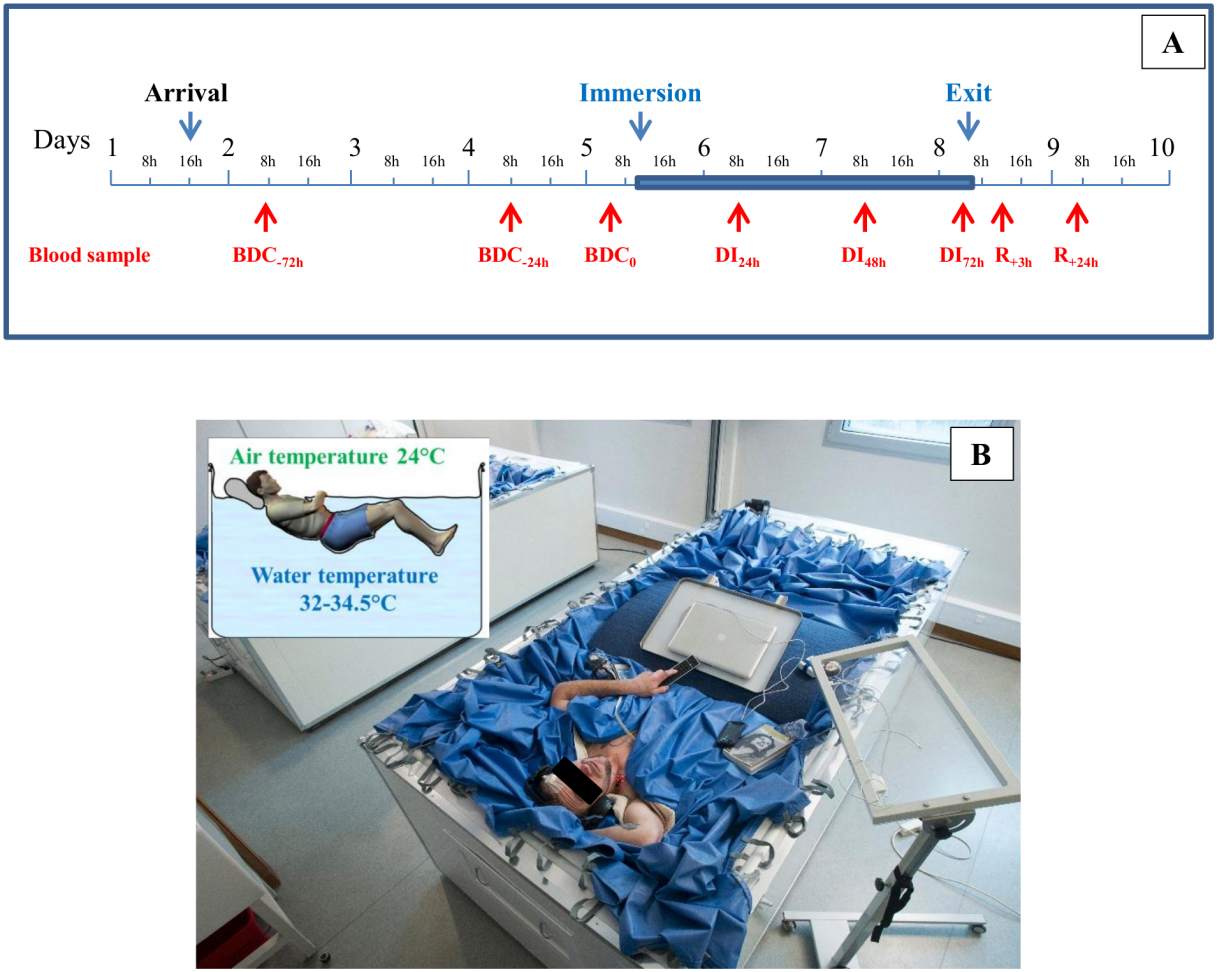
Description of the study. **A** Time line of the experiment with the timing of different blood samples. Blue area corresponds to the DI phase. BDC: baseline data collection; DI: dry immersion; R: recovery. **B** A volunteer in the DI bath.

This study was conducted in a period lasting 9 days as described in Fig 1A. The volunteer arrived on day 1 at the end of the afternoon. The study included the following: 3 days for ambulatory control period (BDC: baseline data collection) (BDC_-72h_, BDC_-24h_, BDC_-0_); 3 days of DI (DI_24h_, DI_48h_, DI_72h_); 1 day for recovery after the immersion period (R_+24h_). In the period preceeding and following DI, all subjects remained active and ambulatory. All were asked not to exercise during the 8 days of the experiment. During the DI phase, the subjects remained continuously immersed in a supine position, except for the different protocols and hygienic procedures; this time out of immersion was evaluated to be a total duration of 5 h between BDC_-0_ and exit of immersion; however, during these procedures, subjects were maintained at a 6° head-down position. In DI, only the head and neck were not entirely immersed in water as shown in Fig 1B. The subjects were supervised by medical control and monitored 24 h/day. Subjects received three solid meals/day during the study with the requirement to finish all meals. The individual energy intake was calculated by multiplying resting metabolic rate with a physical activity level of 1.6 during pre- and post-DI and 1.3 during DI. Coffee, tea, alcohol, smoking, and drugs were prohibited throughout the experiment. Only paracetamol was allowed if needed.

### DI design

A description of the DI method was detailed previously [13]. Briefly, the experiments with DI were performed in a specially designed bath filled with tap water (Fig 1B). A special highly elastic waterproof fabric was attached to a metal rim around the external margin of the bath. The area of the fabric considerably exceeded the area of the water surface (if the bath was empty, the fabric would reach the bottom). The bath had a built-in lift for lowering and raising the subject. The subject, dressed in a comfortable sport suit, was placed on the waterproof fabric after the fabric had first been covered with a cotton sheet for hygienic reasons. The subject was lowered slowly into the water on the lift and his body was gradually covered with the folds of fabric together with the water they contained. The fabric was thin and of sufficient area to allow the subject to appear to be ‘‘freely suspended” in the water mass, under conditions that were similar to a complete lack of structural support. Thus, subjects remained immersed in a supine position and were permitted to put their hands out to work with a computer, eat, read, and perform experimental tasks. The water temperature was regulated automatically. It was set to 33±0.5°C (thermoneutral) and was adjusted for comfort within these limits at the subject’s request. The air temperature was approximately 25°C, in order to maintain the heat balance when the subject was to be raised from the bath. The subject remained under constant medical observation 24 h/day. For physical examinations and visual inspections of the skin, the folds of fabric could be moved apart without changing the experimental conditions substantially.

### Blood sampling

Fasting blood samples were collected always at the same time (around 7.30 am) during the course of the study, namely at baseline 3 days (BDC_-72h_), 1 day (BDC_-24h_) and just before the commencement of immersion (BDC_-0_), after 24 h- (DI_24h_), 48 h- (DI_48h_), and 72 h- (DI_72h_) immersion, then after the return to loading conditions, i.e., at 3 h (R_+3h_) and 24 h (R_+24h_) of recovery. Of note, blood at R_+3h_ was sampled after the tilt test which was made at the exit of immersion so that this sample was not made at fasting state. Serum was immediately separated after blood collection on BD Vacutainer^®^ SST^™^ tubes (with clotting activator). BD Vacutainer^®^ EDTA K3 tubes were used for plasma generation. Serum and plasma were frozen at -80°C right away after the centrifugation until analysis. Measurements were carried out simultaneously in all samples at the end of the study.

### Measurements of biological markers

Calcium, phosphorus and 25-hydroxyvitamin D were measured at BDC_-72h_ and at R_+24h_. All other parameters were measured at each BDC timing, at DI_24h_, DI_48h_ and R_+3h_, except for insulin, glucose and leptin which were not measured at R_+3h_ but at DI_72h_. Moreover, leptin, glucose, insuline and ultrasensitive C-reactive protein (usCRP) were also quantified at R_+24h_.

#### Serum samples

C-terminal crosslinked telopeptide of type I collagen [CTx], procollagen type I N-terminal propeptide [P1NP], bone alkaline phosphatase [bAP], intact and N-mid fragment [OC] and insulin-like growth factor 1 [IGF1] were determined by automated chemiluminescence immunoassay (IDS-iSYS automated analyzer, Boldon, UK). The following parameters were measured by enzyme-immunoassay (EIA) kits: N-terminal crosslinked telopeptide of type I collagen [NTx] (Osteomark^®^NTx, Ostex International, Inc., Seattle, WA, USA); tartrate-resistant acid phosphatase isoform 5b [TRAP5b], procollagen type I C-terminal propeptide [P1CP] and sclerostin [Sost] (Quidel Corporation San Diego, CA, USA); undercarboxylated [Glu-OC] and carboxylated [Gla-OC] OC (Takara Bio, Inc., Otsu, Japan); dickkopf-1 [DKK1] (Biomedica, Wien, Austria); secretory form of nicotinamide phosphoribosyl-transferase [visfatin] (Adipogen AG, Liestal, Switzerland). Serum intact parathyroid hormone [1–84 PTH] was measured using electrochemiluminescence immunoassay (Cobas^®^8000 modular analyzer, Roche Diagnostics Ltd., Rotkreuz, Switzerland). Calcium and phosphorus were quantified using photometric, potentiometric and turbidimetric methods (Architect c8000 clinical chemistry analyzer, Abbott Park, Illinois, USA). 25-hydroxyvitamin D was measured using chemiluminescence immunoassay (Architect i2000SR chemiflex analyzer, Abbott Park, Illinois, USA). Insulin and leptin were evaluated using the Architect c16000 automated clinical chemistry analyzer (Abbott Park, Illinois, USA).

#### Plasma samples

The following parameters were measured also by EIA kits: osteopontin [OPN] (Immuno-Biological Laboratories Co. Ltd, Gunna, Japan); osteoprotegerin [OPG] and receptor activator of nuclear factor kappa β ligand [RANKL] (Biomedica, Wien, Austria); adiponectin (TECOmedical AG, Sissach, Switzerland); neutrophil gelatinase-associated lipocalin [lipocalin-2] (Epitope Diagnostics, Inc., San Diego, CA, USA); Fibroblast Growth Factor 23 [FGF23] (Immutopics, Inc., San Clemente, CA, USA); proinflammatory cytokines [IL-1α, IL-6, IL-17 and TNFα] (R&D systems, Inc., Minneapolis, USA). Glucose and usCRP were evaluated using the Architect c16000 automated clinical chemistry analyzer (Abbott Park, Illinois, USA).

### Statistical analysis

For each parameter, baseline level (BDC) was calculated as the mean value of the measured variables at BDC_-72h_, BDC_-24h_ and BDC_-0_. Data were expressed as median and interquartile range (IQR). The change from baseline (BDC), at DI_24h_ to DI_72h_ or R_+3h_ and/or R_+24h_ for all parameters, was expressed as the percent difference from baseline. The statistical significance of the change with respect to baseline was assessed using non parametric Friedman rank-sum test. Wilcoxon tests corrected by the fdr adjustment method of Benjamini & Hochberg [14] were used for post hoc comparisons. The relationships between changes in parameters at DI_48h_ (expressed vs. BDC) were investigated using nonparametric Spearman correlations. All statistical tests were carried out with the R statistic software supported by the R Foundation for Statistical Computing. P values less than 0.05 were considered to be statistically significant.

## Results

### Body weight

Body weight was found significantly decreased after 1 day immersion and remained so during all the immersion period (74.7±2.1 kg on BDC vs. 73.4±2.0 kg on DI_24h_ and 73.2±2.0 kg on DI_72h_, p = 0.005 and 0.006, respectively). Body weight did not fully normalize after 2 days reloading (74.1±2.1 kg on R_+48h_, p = 0.005 vs. BDC).

### Bone metabolism

Baseline data and measurements of different markers at each point of the immersion period are summarized in Tables 1 and 2.

**Table 1:** Serum markers for different metabolic pathways during the 3-day dry immersion and/or after 24 h- recovery.

	BDC	DI_24h_	DI_48h_	DI_72h_	R_+24h_
**Markers for phosphocalcic metabolism**					
Calcium (mg/L)	94.5(92.8–96.0)				94.0(92.8–97.0)
Phosphorus (mg/L)	37.5(35.5–41.5)				39.0(36.8–41.0)
25OH-D (ng/mL)	19.5(15.0–26.8)				20.5(15.8–26.5)
**Markers for insulino-resistance**					
Glucose (mmol/L)	4.9(4.8–5.0)	5.2(4.8–5.4)	5.0(4.7–5.1)	4.8(4.7–5.0)	4.9(4.7–4.9
Insulin (μU/mL)	4.9(4.0–6.1)	6.4(5.4–7.0) ^a^	5.6(5.0–6.7)	7.3(5.7–7.8) ^a^	6.7(5.2–7.8) ^a^
**Marker for adipocyte activity**					
Leptin (ng/mL)	1.63(1.29–2.86)	1.84(1.58–3.06)	1.82(1.15–3.06)	1.75(1.57–3.32) ^a^^,^^c^	1.44(1.23–2.73) ^d^
**Markers for Inflammatory response**					
usCRP (mg/L)	0.40(0.3–0.5)	0.35(0.3–0.7)	0.50(0.2–0.8)	0.55(0.4–0.8)	0.55(0.3–0.8)

Measurements were made at baseline (BDC), during dry immersion (DI) and after recovery. Data are expressed as median(interquartile range) for n = 12.

^a,b,c,d^: significantly different (p<0.05) vs. BDC, DI_24h_, DI_48h_, and DI_72h_, respectively.

25OH-D: 25-hydroxyvitamin D; usCRP: ultrasensitive C-reactive protein.

**Table 2:** Serum markers for bone turnover and bone metabolism regulators during the 3-day dry immersion and after 3 h- recovery.

	BDC	DI_24h_	DI_48h_	R_+3h_^†^
**Markers for bone turnover**				
***Resorption activity***				
TRAP5b (U/L)	3.37(2.58–4.48)	3.62(2.71–4.61) ^a^	3.56(2.85–4.81) ^a^	3.56(2.89–4.46) ^a^
CTx (pmol/L)	6658(4857–9560)	6971(4968–9862)	7192(5739–11138) ^a^	3178(2260–3696) ^a^^,^^b^^,^^c^
NTx* (nmolBCE/L)	19.0(16.5–21.9)	20.0(17.5–22.6) ^a^	21.6(17.3–26.0) ^a^	15.6(13.5–17.7) ^a^^,^^b^^,^^c^
***Formation activity***				
P1CP (μg/L)	158.1(135.6–231.9)	157.7(115.0–197.1) ^a^	152.0(111.3–181.8) ^a^^,^^b^	127.9(99.8–169.6) ^a^^,^^b^^,^^c^
P1NP (μg/L)	74.9(56.2–89.2)	70.5(51.0–78.4) ^a^	70.3(49.8–80.3) ^a^	66.0(48.1–76.0) ^a^^,^^b^^,^^c^
bAP (μg/L)	16.5(12.1–19.8)	18.6(13.9–21.7) ^a^	18.7(14.9–22.2) ^a^	18.9(15.5–22.4) ^a^
OPN (ng/mL)	467.6(422.0–520.9)	455.2(402.2–502.5)	480.7(397.8–593.5)	509.4(416.8–562.0)
OPG (pmol/L)	3.35(2.68–3.65)	3.25(2.39–3.44)	2.99(2.65–3.40) ^a^	3.28(2.81–3.31)
RANKL (pmol/L)	0.16(0.12–0.24)	0.12(0.07–0.17)	0.07(0.05–0.15) ^a^^,^^b^	0.06(0.05–0.13) ^a^^,^^b^^,^^c^
RANKL/OPG	0.05(0.04–0.08)	0.04(0.02–0.07)	0.03(0.02–0.06) ^a^	0.02(0.01–0.06) ^a^
Intact OC (ng/mL)	22.1(19.4–24.8)	23.2(18.1–24.6)	21.0(19.3–23.9)	19.9(14.8–22.1) ^a^^,^^b^^,^^c^
Gla-OC (ng/mL)	14.0(11.1–16.3)	13.2(11.3–16.4)	12.3(10.5–15.9) ^a^^,^^b^	9.9(8.9–12.7) ^a^^,^^b^^,^^c^
Glu-OC (ng/mL)	6.3(4.7–8.4)	6.6(5.2–10.6)	6.6(6.3–10.4) ^a^	6.4(6.0–10.8) ^a^
Glu-/Gla-OC	0.51(0.30–0.66)	0.59(0.35–0.81)	0.69(0.42–0.83) ^a^^,^^b^	0.75(0.59–0.84) ^a^^,^^b^^,^^c^
***Osteocyte activity***				
DKK1 (pmol/L)	51.7(40.4–64.2)	57.7(42.5–67.5)	59.4(48.3–68.6)	55.6(47.0–69.4)
Sclerostin (ng/mL)	0.40(0.38–0.45)	0.45(0.37–0.47)	0.43(0.39–0.47)	0.41(0.35–0.47)
**Regulators for phosphocalcic metabolism**				
FGF23 (pg/mL)	42.6(33.3–46.3)	32.9(23.5–46.2)	42.4(33.7–48.8) ^b^	44.3(39.1–50.6) ^b^
PTH (ng/L)	20.7(20.1–29.4)	21.0(19.8–27.2)	19.9(16.8–27.4) ^a^	23.5(15.4–28.2)
**Metabolism regulators**				
Adiponectin (μg/mL)	5.1(3.1–7.6)	5.3(2.8–7.3)	3.8(2.1–5.4) ^b^	3.9(2.8–7.0)
Visfatin (ng/mL)	4.0(1.9–5.2)	7.4(3.8–10.1) ^a^	6.1(4.2–9.6) ^a^	6.9(3.0–10.3) ^a^
Lipocalin-2 (ng/mL)	139.7(123.3–143.1)	133.3(126.0–137.7)	133.6(125.3–140.0)	146.8(139.7–150.4) ^b^^,^^c^
IGF1 (μg/L)	203.0(182.7–214.0)	225.3(205.5–234.8) ^a^	230.3(211.1–245.1) ^a^^,^^b^	234.8(209.6–252.4) ^a^^,^^b^

Measurements were made at baseline (BDC), during dry immersion (DI) and after recovery. Data are expressed as median (interquartile range) for n = 12 except for NTx* which was made for n = 6 only.

^a,b,c^: significantly different (p<0.05) vs. BDC, DI_24h_, DI_48h_, respectively.

^†^Of note R_+3h_ is not at fasting state.

TRAP5b: tartrate-resistant acid phosphatase isoform 5b; CTx: C-terminal crosslinked telopeptide of type I collagen; NTx: N-terminal crosslinked telopeptide of type I collagen; P1CP: procollagen type I C-terminal propeptide; P1NP: procollagen type I N-terminal propeptide; bAP: bone alkaline phosphatase; OPN: osteopontin; OPG: osteoprotegerin; RANKL: receptor activator of nuclear factor kappa β ligand; OC: osteocalcin; DKK1: dickkopf-1; FGF23: fibroblast growth factor 23; PTH: parathyroid hormone; IGF1: insulin growth factor 1.

#### Phosphocalcic metabolism

No difference in calcemia and phosphatemia were observed between BDC and R_+24h_. Also, serum 25-hydroxyvitaminD levels were unchanged between BDC and R_+24h_. Serum intact PTH levels decreased during DI and its values became significantly lower at DI_48h_ by 11%. Serum FGF23, a major regulator of phosphate excretion, was not significantly challenged under DI but a trend towards decrease was seen at DI_24h_ leading to obtain values at this timing to be significantly different vs. DI_48h_ and R_+3h_.

#### Bone resorption activity

**Fig 2:**
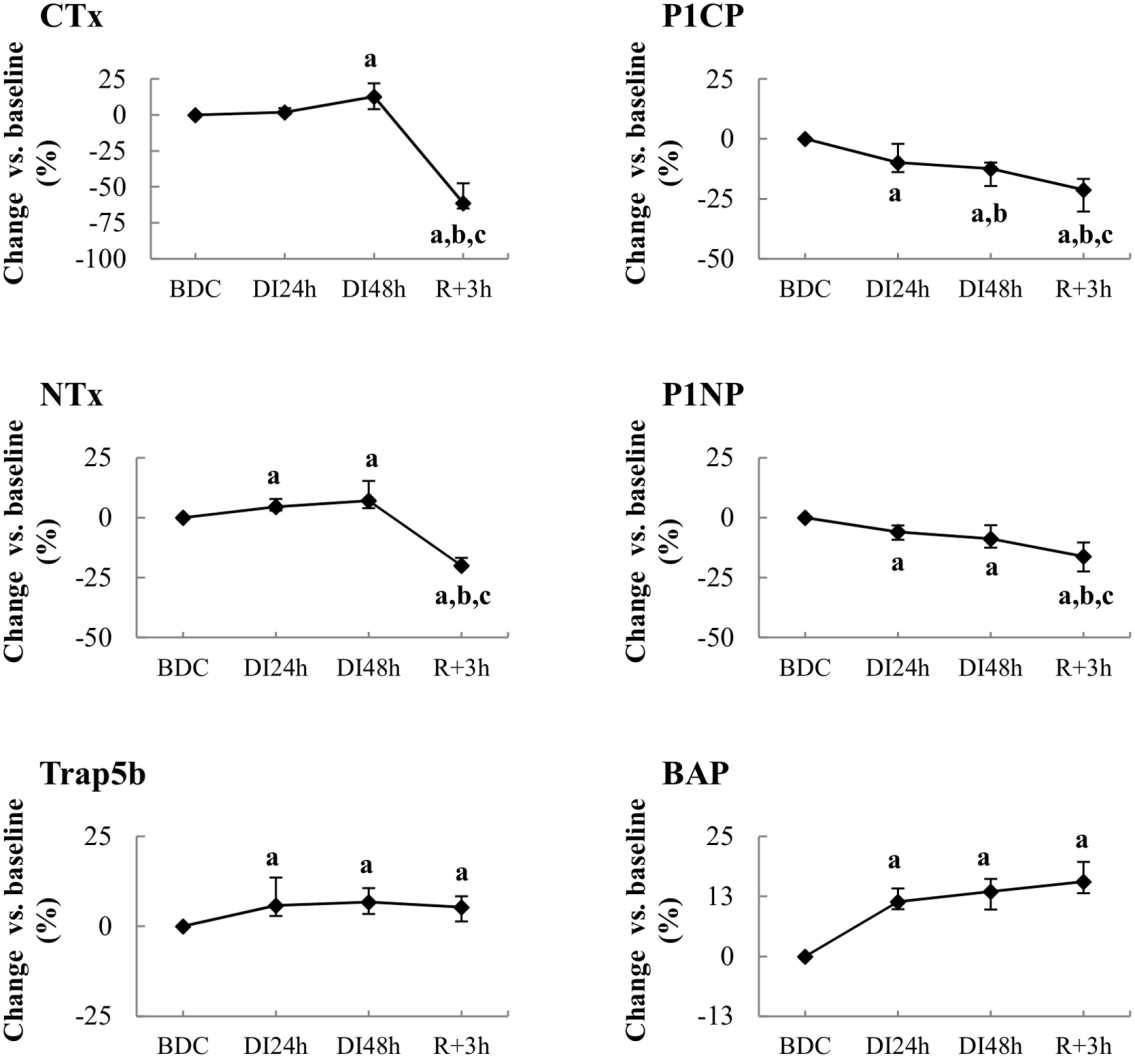
Effect of dry immersion on bone metabolism. Changes in bone resorption (at left) and formation or mineralization (at right) markers during dry immersion. Values are expressed as medians ± interquartile range for n = 12 except for NTx* which was made for n = 6 only. **a,b,c** indicate significant differences vs. BDC, DI_24h_ and DI_48h_, respectively. CTx: C-terminal crosslinked telopeptide of type I collagen; NTx: N-terminal crosslinked telopeptide of type I collagen; TRAP5b: tartrate-resistant acid phosphatase isoform 5b; P1CP: procollagen type I C-terminal propeptide; P1NP: procollagen type I N-terminal propeptide; bAP: bone alkaline phosphatase.

Serum NTx and TRAP5b significantly increased as soon as the first day of immersion and reached 7% higher levels at DI_48h_ compared to BDC (Fig 2). Serum CTx increased later, becoming significantly higher by 13% after 2 days immersion. At return to loading conditions (R_+3h_), CTx and NTx levels decreased below BDC; at the same time, elevated TRAP5b activity was maintained.

#### Bone formation activity

P1CP and P1NP decreased progressively during all immersion periods reaching significantly lower levels by 12% and 9% at DI_48h_ and 21% and 16%, at reambulation, respectively (Fig 2). Serum bAP concentrations increased early by 11% as soon as 1-day immersion and remained elevated during all the course of study (14% at DI_48h_ and 16% at R_+3h_). Serum OPN was unaltered by immersion conditions.

Circulating serum levels of both OPG and RANKL were found significantly decreased by 8% and 45% at DI_48h_, respectively. This induced a decrease in RANKL/OPG ratio of 37% at DI_48h_ and 50% and R_+3h_ vs. BDC.

**Fig 3:**
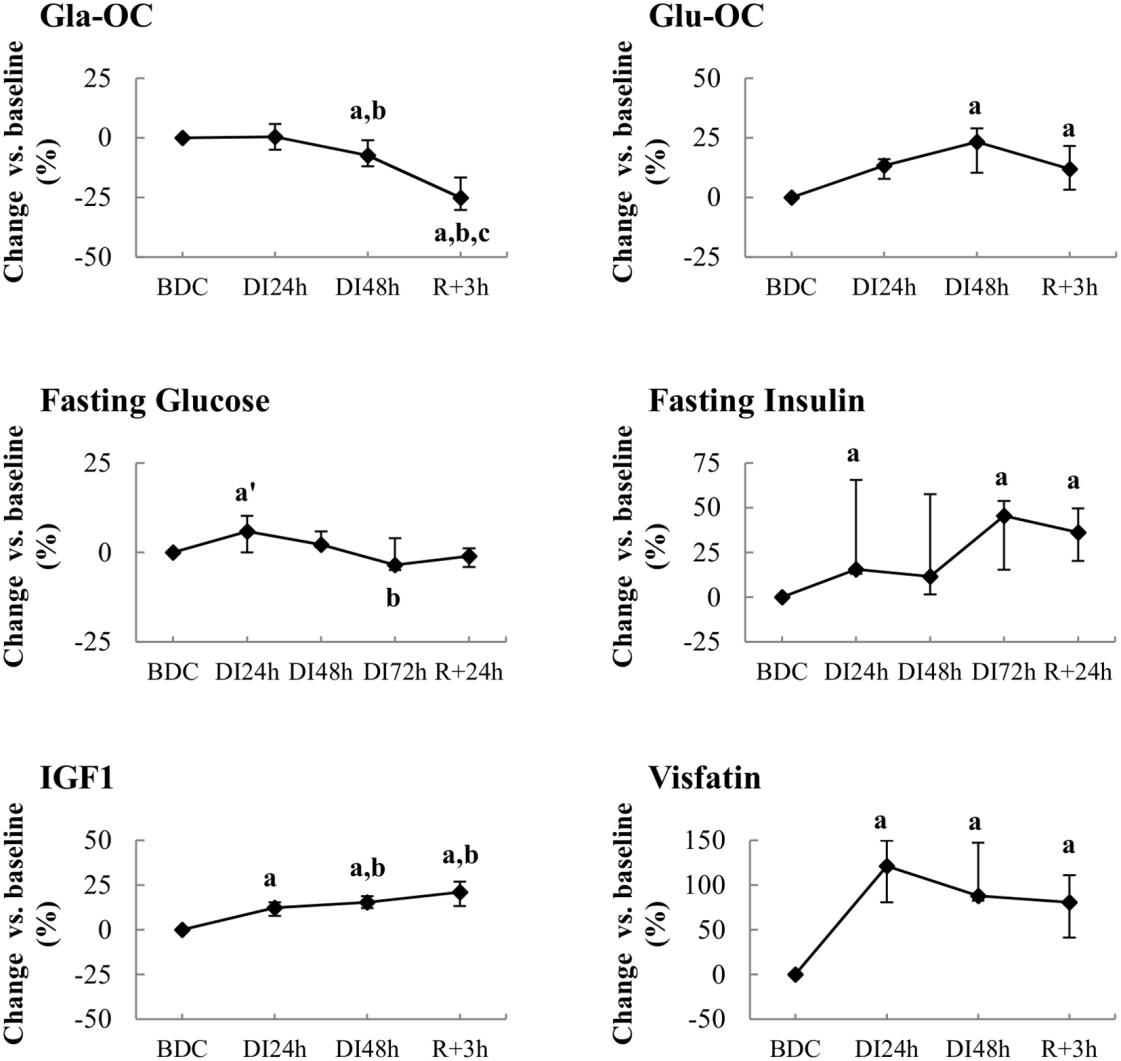
Effect of dry immersion on the different forms of osteocalcin and on the insulinic response. *At Top*, changes in carboxylated (Gla-OC) and decarboxylated (Glu-OC) forms of osteocalcin during dry immersion. *At middle*, changes in values of glucose and insulin during dry immersion. *At bottom*, changes in values of IGF1 and visfatin during dry immersion. Values are medians ± interquartile range for n = 12. **a,b,c** indicate significant differences vs. BDC, DI_24h_ and DI_48h_, respectively; **a’** different vs. BDC p<0.10.

Total intact OC level was unchanged during the immersion phase. Nevertheless, a slight decrease in its carboxylated forms (Gla-OC) and an increase in its undercarboxylated forms (Glu-OC) were observed (Fig 3). At reambulation, while Glu-OC level maintained its elevated level, circulating Gla-OC concentrations continued to decline, thus leading to a significant fall in total OC by 14% compared with BDC. These changes induced a progressive increase in Glu/Gla-OC ratio during all the study.

#### Osteocyte activity

Sclerostin and DKK1, Wnt signaling antagonists produced predominantly by osteocytes and considered to be inhibitors of osteoblast activity, were unaltered by immersion conditions (Table 2).

### Energy metabolism and hormones levels

In spite of a significant decrease between DI_24h_ and DI_48h_, adiponectemia was not clearly affected by immersion conditions when compared to BDC. Circulating leptin concentrations increased under immersion so that levels increased significantly at DI_72h_. Lipocalin-2, a potential regulator of bone homeostasis, was found higher by 16% at R_+3h_ compared to DI_24h_. Visfatin concentrations almost doubled after 1 day of immersion and remained elevated (Fig 3). Fasting glucose levels tended to increase (p<0.10) only after 24 h immersion; then they returned to BDC values (Fig 3). At the same time, fasting insulin concentrations increased during all the DI phase. Furthermore, circulating IGF1 levels progressively increased during immersion. At the systemic level, no inflammation was observed as assessed by unchanged usCRP values and undetectable levels in inflammatory cytokines (IL-1β, IL-6, TNFα, IL-17).

### Associations between biochemical blood markers

**Fig 4:**
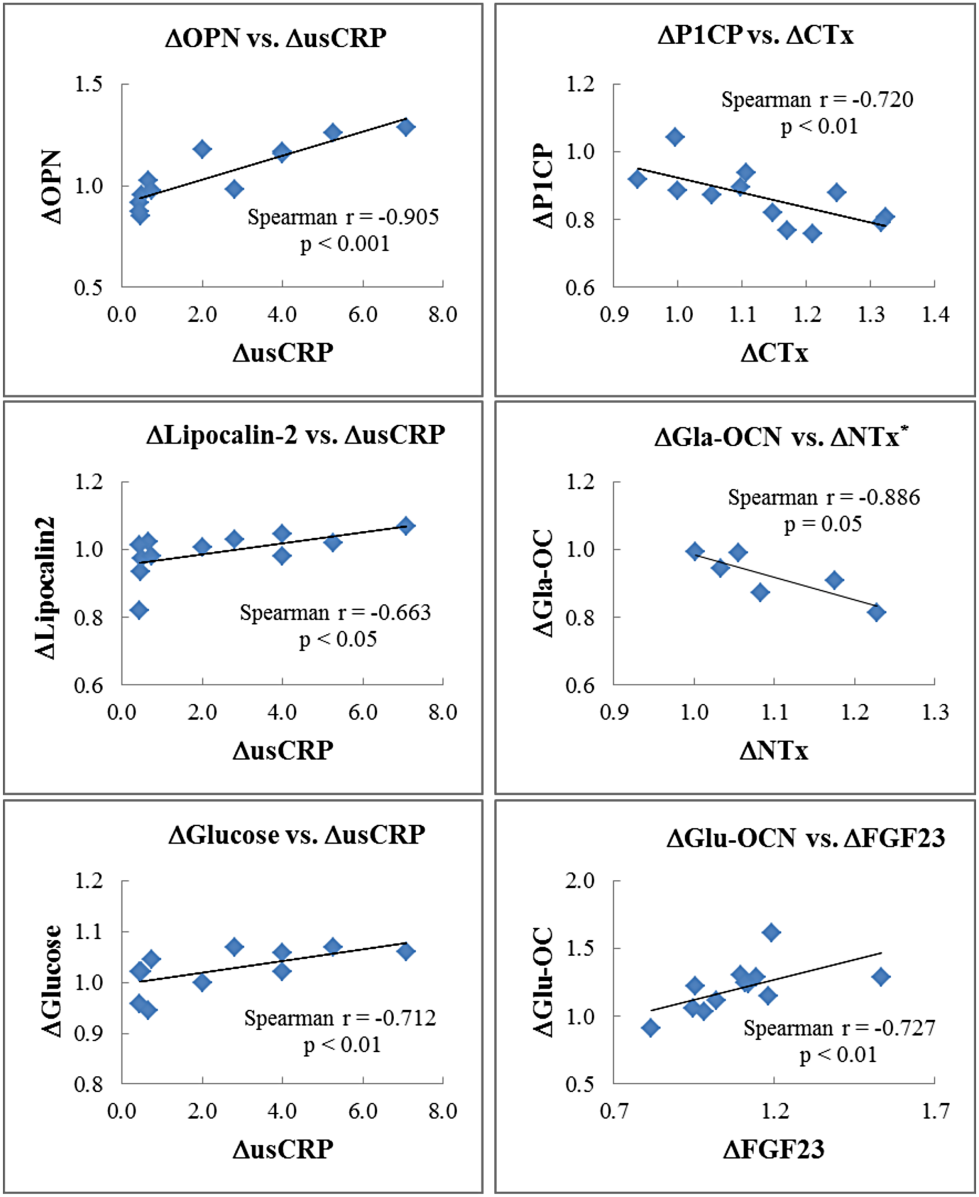
Association between biochemical blood markers. Spearman correlations between percent changes at DI_48h_ in bone and energy metabolism parameters for n = 12 except for NTx* which was made for n = 6 only. Changes are expressed in percent from BDC. OPN: osteopontin; usCRP: ultrasensitive C-reactive protein; P1CP: procollagen type I C-terminal propeptide; CTx: C-terminal crosslinked telopeptide of type I collagen; OC: osteocalcin; Gal-OC and Glu-OC: carboxylated and uncarboxlalted forms of OC, respectively; NTx: N-terminal crosslinked telopeptide of type I collagen; usCRP: ultrasensitive C-reactive protein; FGF23: fibroblast growth factor 23.

Significant correlations were detected between changes in blood markers measured at DI_48-h_ (Fig 4). Firstly, percent changes of usCRP positively correlated with changes of OPN, lipocalin-2 and fasting glucose. Furthermore, a positive correlation was established between changes in FGF23 and Glu-OC. Lastly, changes in bone resorption markers (CTx or NTx) negatively correlated with changes in bone formation markers (P1CP or Gla-OC).

## Discussion

This study provided a biochemical assessment of adaptation to a human unloading model to investigate some of the effects of space-flight without the limitations of spaceflight itself. DI is a ground-based model of prolonged conditions of microgravity previously used in Russia. It has been recently implemented in France (CNES and MEDES) and we are reporting here results of the first campaign. As it is expected to induce severe discomfort [13], this first experiment was planned for a short duration of 3 days.

### Body weight

The decrease in body mass of near 1.5 kg confirms the result already reported in previous studies of DI [15,16]. It was similar to the loss observed after ten days of bed rest [17]. This loss of weight is coherent with the “relative dehydrated status” induced by these 3 days in immersion conditions leading to a 17% decrease in plasma volume as reported by Treffel et al. [18]. Surprisingly, protein concentrations in the plasma did not increase during DI despite the reduction in plasma volume. As well discussed by Navasiolava et al. [13], this might be explained by the partial transfer of plasma proteins to the interstitial fluid due to an increased transcapillary escape rate and decreased lymphatic return.

### Bone remodeling activity

Markers of bone tissue resorption such as TRAP and CTx were shown to slightly increase during Russian DI for 7 days [19]. However, Markin et al. [20] did not find any changes in the activity of total acid phosphatase during a similar period of immersion. From urinary NTx and Deoxypyridinoline (Dpyr) as bone turnover markers, previous reports have shown that already 24 hours of bed rest induce a significant rise in resorption activity in healthy subjects [3,4]. However, when resorption activity is evaluated from blood analysis, resorption markers measured from the 5^th^ day of bed rest became significantly increased only beyond 10 days [21]. Our results showed that circulating levels of both TRAP5b and NTx were already elevated as early as the first day of immersion and during all the immersion phase. CTx was also elevated at DI_48h_. Thus, DI has an earlier effect than bed rest on bone resorption. The substantial decrease of RANKL leading to decreased RANKL/OPG ratio at DI_48h_ was unexpected in view of increased bone resorption markers. However, circulating RANKL is likely to be influenced by a variety of non-osseous processes including inflammatory, infectious and immunological processes [22,23].

In our study, CTx and NTx levels measured at R_+3h_ greatly decreased compared to BDC. A similar observation was made for OC level at R_+3h_. These results might not be solely related to immersion condition and/or recovery but also to the fact than blood sampling at this timing was not made at fasting, a parameter known to affect bone marker circadian variation [24,25].

In our study, elevated bone resorption was associated with a decrease in markers of bone formation (P1NP, P1CP) whereas bAP increased after 24- h DI. A lack of changes in P1NP and bAP was reported by Kopp [19] after 5 and 7 days of immersion. Our changes in bAP are in agreement with studies of unloading by experimental bed rest, which have also shown an early increase of its activity suggesting an enhanced mineralization of the collagenous matrix [21,26,27]. On the other hand, data from bed rest studies are discordant with regard to bone formation parameters. Indeed, P1NP levels usually display either no obvious changes or slight decreases. Thus, reduced P1NP concentrations were reported after 6 to 11 days and maintained reduced during 21 days of bed rest [27,28]. In contrast, Belavy et al. [21] did not find any change in P1NP level during 60 days of bed rest. Also no change in P1CP was observed during 42 days of bed rest by Uebelhart et al. [29].

In summary, our findings show that DI challenged bone remodeling activity more dramatically than the head-down bed rest, possibly related to the lack of body support and proprioceptive deprivation in DI. This unbalance between bone cellular activities supports the rapid onset of bone alterations as reported by Kotov et al. [30]. These authors observed that, after 7 days of DI, the bone mineral density of the lower part of the skeleton (proximal epiphysis of the femur) tends to be approximately 2% lower and that of the upper part (skull, hand, costal bones) tends to be 2% higher when compared to baseline.

### Bone remodeling and energy metabolism

Blood glucose concentration increased after 24- h DI, then it normalized to baseline level after 48- h DI. At the same time, we could observe higher blood levels of both insulin and IGF1. These results suggest an activation of hepatic metabolism via the stimulation of growth hormone as such findings were already reported in previous DI studies [20,31]. This favors the hypothesis that insulin resistance develops during DI, similarly to that observed after 6 days of head-down bed rest [32]. As shown by Karsenty’s group, insulin was identified as a key molecular link between bone remodeling and energy metabolism. Indeed, insulin signaling in osteoblasts limits production of OPG, an inhibitor of osteoclast maturation [33,34]. In our study, decreased OPG and increased bone resorption activity can be related to increasing insulin signaling. Bone resorption occurs at acid pH enough to decarboxylate proteins; thus osteoclasts determine the carboxylation status and function of OC. Lee et al. [7] reported that Glu-OC was the active form of the molecule in rodent models. In our study, all forms of this molecule (i.e. Glu-OC, Gla-OC and total intact OC) have been measured; this allowed us to evidence that DI does not change circulating OC but increased the proportion of Glu-OC released by the osteoclasts as a result of enhanced resorption activity. Noteworthy, we found a positive relationship between changes of Glu-OC and FGF23, the two main osteoblast/cyte hormones. These hormones are regulated by bone remodeling and mineralization per se, and by other hormones such as leptin, insulin, glucocorticoids, PTH, and vitamin D, leading to complex cross-talk that begun to be decrypt [35]. Rare are the studies which have measured Glu-OC under unloading models. Morgan et al. [5] reported no change in circulating Glu-OC whereas bone resorption markers were greatly increased by 80% after 30-days head-down bed rest. Fernandez-Real et al [36] established a positive association between circulating OC and insulin sensitivity, secretion and disposition index (r = 0.41, p<0.05) among lean men. In our study, no relationship was able to be established between these different serum markers. However the concomitant increases in Glu-OC and insulin under our immersion conditions seems to be in agreement with studies suggesting that OC might be an active regulator of insulin sensitivity. This could confirm the role of bone as an endocrine organ in humans. Surprisingly, increased circulating IGF1 was associated with decreased bone formation markers although this skeletal growth factor is known to stimulate bone formation. Such a finding leads to suppose the development of resistance to this skeletal growth factor as discussed by Bikle et al. [37] in rats submitted to a hindlimb unloading model. Indeed, these authors evidenced the failure of the unloaded bone to grow in response to exogenous IGF1.

In our study, no inflammatory response has been evidenced. However, percent changes of usCRP correlated positively with changes of OPN, lipocalin-2 and fasting glucose. In addition to its functions in biomineralization, OPN also acts as a cytokine that takes part in the inflammatory response [38]. Abundant evidence suggests that OPN plays a pivotal role in the development of adipose tissue inflammation and insulin resistance [39,40]. Furthermore, a role of lipocalin-2 in energy metabolism and inflammation has been shown [41–43]. Lipocalin-2 is expressed by osteoblasts at levels tenfold higher than in adipose tissue or other organs and was recently identified as an additional bone hormone able to suppress appetite through binding to melanocortin 4 receptor [44]. This is probably why its level is increased at R_+3h_, soon after a meal (while DI_24h_ and DI_48h_ were in fasting state). Lipocalin-2 was also identified as a novel osteoblast mechanoresponding gene with a role in bone homeostasis. It was found upregulated in osteoblasts in a manner proportional to the reduction of gravitational force intensity [45], and in long bones of various murine models of mechanical unloading [46]. In human subjects submitted to bed-rest conditions, lipocalin2 serum level increased from 12th days suggesting that it is a late marker of unloading [46].

On the other hand, 3-days of DI induced an almost two-fold increase in visfatin concentrations. This molecule is synthesized by the visceral adipose tissue; it exists in an intracellular form acting as nicotinamide phosphoribosyltransferase in nicotinamide adenine dinucleotide biosynthesis, and an extracellular form which is an insulin-mimetic, pro-inflammatory/immune-modulating adipokine [47]. Its circulating levels, although with some concerns [48], are associated with obesity, insulin resistance and energy-bone crosstalk [49]. To our knowledge, very few studies investigated this adipokine under ground-based models of prolonged conditions of microgravity. The effects of bed rest on serum adipokines and low-grade inflammation were shown to be age-dependent [50]. Indeed, in this latter study, young subjects (18–30 years) responded to 14-days bed rest by increasing serum visfatin and resistin while there was no difference in older adults (53–65 years), these latter subjects increasing rather in IL-6 and TNF-α. Thus, the effects of DI in our subjects between 26 and 39 years old were similar to those of bed rest when we consider subjects of the same age range. Although visfatin was originally thought to have insulin mimetic effects by binding and activating insulin receptor, subsequent studies have been unable to repeat those results and thus have been retracted [51]. In our study, no statistical correlation was established between changes in insulin and visfatin. As discussed by Rudwill et al. [52], visfatin remains a controversial molecule, whose role and regulation still need to be defined. These authors proposed a regulation of visfatin by physical activity, independent of measurable changes in energy balance, as visfatin circulating levels in fasting state increased under physical inactivity conditions. Despite these uncertainties, the present study revealed a great responsiveness for this parameter that might constitute an early marker of unloading conditions in humans.

## Conclusion

The microgravity simulated model by short-term DI is able to challenge bone remodeling activity more dramatically than the head-down-tilt bed rest. Enhanced insulin response showed a beginning of insulin-resistance, which was concomitant to a higher degree of OC decarboxylation. This latter finding confirms the role of bone as an endocrine organ regulating energy metabolism in humans. Furthermore, visfatin for which a great responsiveness was observed in our study could represent a very interesting marker to analyze early events of unloading in humans.

## Supporting information

S1 File Avis favorable ansm. (PDF)

S2 File Avis favorable CPP immersion seche. (PDF)

S3 File Supporting information files for PLOS One. (XLSX)

## References

[1] Nagaraja MP, Risin D. The current state of bone loss research: data from spaceflight and microgravity simulators. J Cell Biochem. 2013;114: 1001–1008. doi: 10.1002/jcb.24454 23150462 10.1002/jcb.24454

[2] Hargens AR, Vico L. Long-duration bed rest as an analog to microgravity. J Appl Physiol Bethesda Md 1985. 2016;120: 891–903. doi: 10.1152/japplphysiol.00935.2015 26893033 10.1152/japplphysiol.00935.2015

[3] Heer M, Baecker N, Mika C, Boese A, Gerzer R. Immobilization induces a very rapid increase in osteoclast activity. Acta Astronaut. 2005;57: 31–36. 15900645 10.1016/j.actaastro.2004.12.007

[4] Baecker N, Tomic A, Mika C, Gotzmann A, Platen P, Gerzer R, et al Bone resorption is induced on the second day of bed rest: results of a controlled crossover trial. J Appl Physiol Bethesda Md 1985. 2003;95: 977–982. doi: 10.1152/japplphysiol.00264.2003 12909597 10.1152/japplphysiol.00264.2003

[5] Morgan JLL, Zwart SR, Heer M, Ploutz-Snyder R, Ericson K, Smith SM. Bone metabolism and nutritional status during 30-day head-down-tilt bed rest. J Appl Physiol Bethesda Md 1985. 2012;113: 1519–1529. doi: 10.1152/japplphysiol.01064.2012 22995395 10.1152/japplphysiol.01064.2012 PMC3524659

[6] Stettler R, Ith M, Acheson KJ, Décombaz J, Boesch C, Tappy L, et al Interaction between dietary lipids and physical inactivity on insulin sensitivity and on intramyocellular lipids in healthy men. Diabetes Care. 2005;28: 1404–1409. 15920059 10.2337/diacare.28.6.1404

[7] Lee NK, Sowa H, Hinoi E, Ferron M, Ahn JD, Confavreux C, et al Endocrine regulation of energy metabolism by the skeleton. Cell. 2007;130: 456–469. doi: 10.1016/j.cell.2007.05.047 17693256 10.1016/j.cell.2007.05.047 PMC2013746

[8] Schwetz V, Pieber T, Obermayer-Pietsch B. The endocrine role of the skeleton: background and clinical evidence. Eur J Endocrinol. 2012;166: 959–967. doi: 10.1530/EJE-12-0030 22436399 10.1530/EJE-12-0030

[9] Kuro-O M. The FGF23 and Klotho system beyond mineral metabolism. Clin Exp Nephrol. 2017;21: 64–69. doi: 10.1007/s10157-016-1357-6 27838783 10.1007/s10157-016-1357-6

[10] Grethen E, Hill KM, Jones R, Cacucci BM, Gupta CE, Acton A, et al Serum leptin, parathyroid hormone, 1,25-dihydroxyvitamin D, fibroblast growth factor 23, bone alkaline phosphatase, and sclerostin relationships in obesity. J Clin Endocrinol Metab. 2012;97: 1655–1662. doi: 10.1210/jc.2011-2280 22362819 10.1210/jc.2011-2280 PMC3339883

[11] Wojcik M, Janus D, Dolezal-Oltarzewska K, Drozdz D, Sztefko K, Starzyk JB. The association of FGF23 levels in obese adolescents with insulin sensitivity. J Pediatr Endocrinol Metab JPEM. 2012;25: 687–690. doi: 10.1515/jpem-2012-0064 23155694 10.1515/jpem-2012-0064

[12] Lombardi G, Corsetti R, Lanteri P, Grasso D, Vianello E, Marazzi MG, et al Reciprocal regulation of calcium-/phosphate-regulating hormones in cyclists during the Giro d’Italia 3-week stage race. Scand J Med Sci Sports. 2014;24: 779–787. doi: 10.1111/sms.12080 23647316 10.1111/sms.12080

[13] Navasiolava NM, Custaud M-A, Tomilovskaya ES, Larina IM, Mano T, Gauquelin-Koch G, et al Long-term dry immersion: review and prospects. Eur J Appl Physiol. 2011;111: 1235–1260. doi: 10.1007/s00421-010-1750-x 21161267 10.1007/s00421-010-1750-x

[14] Benjamini Y, Hochberg Y. "Controlling the false discovery rate : A practical and Powerful approach to multiple testing. J R Stat Soc Ser B. 1995;57: 289–300.

[15] Iwase S, Sugiyama Y, Miwa C, Kamiya A, Mano T, Ohira Y, et al Effects of three days of dry immersion on muscle sympathetic nerve activity and arterial blood pressure in humans. J Auton Nerv Syst. 2000;79: 156–164. 10699647 10.1016/s0165-1838(99)00076-4

[16] Navasiolava NM, Pajot A, Gallois Y, Pastushkova LK, Kulchitsky VA, Gauquelin-Koch G, et al NT-ProBNP levels, water and sodium homeostasis in healthy men: effects of 7 days of dry immersion. Eur J Appl Physiol. 2011;111: 2229–2237. doi: 10.1007/s00421-011-1858-7 21327795 10.1007/s00421-011-1858-7

[17] Coker RH, Hays NP, Williams RH, Wolfe RR, Evans WJ. Bed rest promotes reductions in walking speed, functional parameters, and aerobic fitness in older, healthy adults. J Gerontol A Biol Sci Med Sci. 2015;70: 91–96. doi: 10.1093/gerona/glu123 25122628 10.1093/gerona/glu123 PMC4342684

[18] Treffel L, Dmitrieva L, Gauquelin-Koch G, Custaud M-A, Blanc S, Gharib C, et al Craniomandibular System and Postural Balance after 3-Day Dry Immersion. PloS One. 2016;11: e0150052 doi: 10.1371/journal.pone.0150052 26913867 10.1371/journal.pone.0150052 PMC4767814

[19] Kopp O. Effects of dry water immersion on bone metabolism (Auswirkungen der trockenen wasserimmersion auf den knochenstoffwechsel). University of Bonn, Germany (in German); 2008.

[20] Markin AA, Morukov BV, Zhuravleva OA, Zabolotskaia IV, Vostrikova LV, Liapunova NA, et al Dynamics of blood biochemical parameters in an experiment with 7-day immersion. Aviaskom Ekol Med. 2008;42(5): 56–59. 19192540

[21] Belavý DL, Baecker N, Armbrecht G, Beller G, Buehlmeier J, Frings-Meuthen P, et al Serum sclerostin and DKK1 in relation to exercise against bone loss in experimental bed rest. J Bone Miner Metab. 2016;34: 354–365. doi: 10.1007/s00774-015-0681-3 26056021 10.1007/s00774-015-0681-3

[22] Findlay DM, Atkins GJ. Relationship between serum RANKL and RANKL in bone. Osteoporos Int J Establ Result Coop Eur Found Osteoporos Natl Osteoporos Found USA. 2011;22: 2597–2602. doi: 10.1007/s00198-011-1740-9 21850548 10.1007/s00198-011-1740-9

[23] Wagner D, Fahrleitner-Pammer A. Levels of osteoprotegerin (OPG) and receptor activator for nuclear factor kappa B ligand (RANKL) in serum: are they of any help? Wien Med Wochenschr 1946. 2010;160: 452–457. doi: 10.1007/s10354-010-0818-x 20714810 10.1007/s10354-010-0818-x

[24] Caillot-Augusseau A, Vergely N, Soler C, Pernot J, Calatoru V, Bruleport V, et al [Study of variations of osteocalcin concentrations in function of collection, assay method and its nyctohemeral rhythm]. Ann Endocrinol. 1995;56: 591–598. 8787350

[25] Qvist P, Christgau S, Pedersen BJ, Schlemmer A, Christiansen C. Circadian variation in the serum concentration of C-terminal telopeptide of type I collagen (serum CTx): effects of gender, age, menopausal status, posture, daylight, serum cortisol, and fasting. Bone. 2002;31: 57–61. 12110413 10.1016/s8756-3282(02)00791-3

[26] Watanabe Y, Ohshima H, Mizuno K, Sekiguchi C, Fukunaga M, Kohri K, et al Intravenous pamidronate prevents femoral bone loss and renal stone formation during 90-day bed rest. J Bone Miner Res Off J Am Soc Bone Miner Res. 2004;19: 1771–1778. doi: 10.1359/JBMR.040811 15476576 10.1359/JBMR.040811

[27] Rittweger J, Debevec T, Frings-Meuthen P, Lau P, Mittag U, Ganse B, et al On the combined effects of normobaric hypoxia and bed rest upon bone and mineral metabolism: Results from the PlanHab study. Bone. 2016;91: 130–138. doi: 10.1016/j.bone.2016.07.013 27443510 10.1016/j.bone.2016.07.013

[28] Frings-Meuthen P, Boehme G, Liphardt A-M, Baecker N, Heer M, Rittweger J. Sclerostin and DKK1 levels during 14 and 21 days of bed rest in healthy young men. J Musculoskelet Neuronal Interact. 2013;13: 45–52. 23445914

[29] Uebelhart D, Bernard J, Hartmann DJ, Moro L, Roth M, Uebelhart B, et al Modifications of bone and connective tissue after orthostatic bedrest. Osteoporos Int J Establ Result Coop Eur Found Osteoporos Natl Osteoporos Found USA. 2000;11: 59–67. doi: 10.1007/s001980050007 10663360 10.1007/s001980050007

[30] Kotov S, Oganov V, Skripnikova I. Imitation of the early effects of weighlessness in human bone tissue in conditions of head down bed rest and dry immersion. Institute of Biomedical Problem, Moscow; 2003.

[31] Afonin BV, Sedova EA. [Digestive system functioning during simulation of the microgravity effects on humans by immersion]. Aviakosmicheskaia Ekol Meditsina Aerosp Environ Med. 2009;43: 48–52. 19462782

[32] Blanc S, Normand S, Pachiaudi C, Fortrat JO, Laville M, Gharib C. Fuel homeostasis during physical inactivity induced by bed rest. J Clin Endocrinol Metab. 2000;85: 2223–2233. doi: 10.1210/jcem.85.6.6617 10852455 10.1210/jcem.85.6.6617

[33] Ferron M, Wei J, Yoshizawa T, Del Fattore A, DePinho RA, Teti A, et al Insulin signaling in osteoblasts integrates bone remodeling and energy metabolism. Cell. 2010;142: 296–308. doi: 10.1016/j.cell.2010.06.003 20655470 10.1016/j.cell.2010.06.003 PMC2910411

[34] Fulzele K, Riddle RC, DiGirolamo DJ, Cao X, Wan C, Chen D, et al Insulin receptor signaling in osteoblasts regulates postnatal bone acquisition and body composition. Cell. 2010;142: 309–319. doi: 10.1016/j.cell.2010.06.002 20655471 10.1016/j.cell.2010.06.002 PMC2925155

[35] Quarles LD. A systems biology preview of the relationships between mineral and metabolic complications in chronic kidney disease. Semin Nephrol. 2013;33: 130–142. doi: 10.1016/j.semnephrol.2012.12.014 23465500 10.1016/j.semnephrol.2012.12.014 PMC5079533

[36] Fernández-Real JM, Izquierdo M, Ortega F, Gorostiaga E, Gómez-Ambrosi J, Moreno-Navarrete JM, et al The relationship of serum osteocalcin concentration to insulin secretion, sensitivity, and disposal with hypocaloric diet and resistance training. J Clin Endocrinol Metab. 2009;94: 237–245. doi: 10.1210/jc.2008-0270 18854399 10.1210/jc.2008-0270

[37] Bikle DD, Harris J, Halloran BP, Morey-Holton ER. Skeletal unloading induces resistance to insulin-like growth factor I. J Bone Miner Res Off J Am Soc Bone Miner Res. 1994;9: 1789–1796. doi: 10.1002/jbmr.5650091116 7532347 10.1002/jbmr.5650091116

[38] Andrews S, Ford D, Martin P. Knockdown of osteopontin reduces the inflammatory response and subsequent size of postsurgical adhesions in a murine model. Am J Pathol. 2012;181: 1165–1172. doi: 10.1016/j.ajpath.2012.06.027 22858059 10.1016/j.ajpath.2012.06.027 PMC3461194

[39] Gómez-Ambrosi J, Catalán V, Ramírez B, Rodríguez A, Colina I, Silva C, et al Plasma osteopontin levels and expression in adipose tissue are increased in obesity. J Clin Endocrinol Metab. 2007;92: 3719–3727. doi: 10.1210/jc.2007-0349 17595250 10.1210/jc.2007-0349

[40] Kiefer FW, Zeyda M, Gollinger K, Pfau B, Neuhofer A, Weichhart T, et al Neutralization of osteopontin inhibits obesity-induced inflammation and insulin resistance. Diabetes. 2010;59: 935–946. doi: 10.2337/db09-0404 20107108 10.2337/db09-0404 PMC2844841

[41] Wang Y, Lam KSL, Kraegen EW, Sweeney G, Zhang J, Tso AWK, et al Lipocalin-2 is an inflammatory marker closely associated with obesity, insulin resistance, and hyperglycemia in humans. Clin Chem. 2007;53: 34–41. doi: 10.1373/clinchem.2006.075614 17040956 10.1373/clinchem.2006.075614

[42] Yan Q-W, Yang Q, Mody N, Graham TE, Hsu C-H, Xu Z, et al The adipokine lipocalin 2 is regulated by obesity and promotes insulin resistance. Diabetes. 2007;56: 2533–2540. doi: 10.2337/db07-0007 17639021 10.2337/db07-0007

[43] Zhang J, Wu Y, Zhang Y, Leroith D, Bernlohr DA, Chen X. The role of lipocalin 2 in the regulation of inflammation in adipocytes and macrophages. Mol Endocrinol Baltim Md. 2008;22: 1416–1426. doi: 10.1210/me.2007-0420 18292240 10.1210/me.2007-0420 PMC2422824

[44] Mosialou I, Shikhel S, Liu J-M, Maurizi A, Luo N, He Z, et al MC4R-dependent suppression of appetite by bone-derived lipocalin 2. Nature. 2017;543: 385–390. doi: 10.1038/nature21697 28273060 10.1038/nature21697 PMC5975642

[45] Capulli M, Rufo A, Teti A, Rucci N. Global transcriptome analysis in mouse calvarial osteoblasts highlights sets of genes regulated by modeled microgravity and identifies a “mechanoresponsive osteoblast gene signature.” J Cell Biochem. 2009;107: 240–252. doi: 10.1002/jcb.22120 19288527 10.1002/jcb.22120

[46] Rucci N, Capulli M, Piperni SG, Cappariello A, Lau P, Frings-Meuthen P, et al Lipocalin 2: a new mechanoresponding gene regulating bone homeostasis. J Bone Miner Res Off J Am Soc Bone Miner Res. 2015;30: 357–368. doi: 10.1002/jbmr.2341 25112732 10.1002/jbmr.2341

[47] Fukuhara A, Matsuda M, Nishizawa M, Segawa K, Tanaka M, Kishimoto K, et al Visfatin: a protein secreted by visceral fat that mimics the effects of insulin. Science. 2005;307: 426–430. doi: 10.1126/science.1097243 15604363 10.1126/science.1097243

[48] Lombardi G, Banfi G. Effects of sample matrix and storage conditions on full-length visfatin measurement in blood. Clin Chim Acta Int J Clin Chem. 2015;440: 140–142. doi: 10.1016/j.cca.2014.11.006 25447705 10.1016/j.cca.2014.11.006

[49] Sommer G, Garten A, Petzold S, Beck-Sickinger AG, Blüher M, Stumvoll M, et al Visfatin/PBEF/Nampt: structure, regulation and potential function of a novel adipokine. Clin Sci Lond Engl 1979. 2008;115: 13–23. doi: 10.1042/CS20070226 19016657 10.1042/CS20070226

[50] Jurdana M, Jenko-Pražnikar Z, Mohorko N, Petelin A, Jakus T, Šimunič B, et al Impact of 14-day bed rest on serum adipokines and low-grade inflammation in younger and older adults. Age Dordr Neth. 2015;37: 116 doi: 10.1007/s11357-015-9848-z 26564239 10.1007/s11357-015-9848-z PMC5005849

[51] Fukuhara A, Matsuda M, Nishizawa M, Segawa K, Tanaka M, Kishimoto K, et al Retraction. Science. 2007;318: 565 doi: 10.1126/science.318.5850.565b 17962537 10.1126/science.318.5850.565b

[52] Rudwill F, Blanc S, Gauquelin-Koch G, Choukèr A, Heer M, Simon C, et al Effects of different levels of physical inactivity on plasma visfatin in healthy normal-weight men. Appl Physiol Nutr Metab Physiol Appl Nutr Metab. 2013;38: 689–693. doi: 10.1139/apnm-2012-0434 23724888 10.1139/apnm-2012-0434

