# Supplementary material for: Effects of short-term dry immersion on bone remodeling markers, insulin and adipokines
Source: PLoS One. 2017 Aug 14;12(8):e0182970. doi: 10.1371/journal.pone.0182970 (PMC5555617; doi:10.1371/journal.pone.0182970)
Supplement: S1 File — (PDF) [file pone.0182970.s001.pdf]

ansm
Agence nationale de sécurité du médicament et des produits de santé

AUTORISATION D'ESSAI CLINIQUE NE PORTANT PAS SUR UN PRODUIT DE SANTE (ESSAI-HPS)

Nombre de pages : 1
(Incluant la page de garde)

Envoi par Télécopie

Date : 3 NOV. 2014

**Identifiants de l'essai clinique**					
Titre	EVALUATION OF THE PHYSIOLOGICAL EFFECTS OF 3 DAYS OF DRY IMMERSION USED AS A GROUND-BASED MODEL TO STUDY THE EFFECTS OF WEIGHTLESSNESS: A PILOT STUDY ON 12 HEALTHY MALE VOLUNTEERS.				
Promoteur	CNES			Réf. CPP	1-14-26
Réf. Promoteur	14-962 / DI study	N° ID RCB	2014-A00904-43	Réf. ANSM	140997B-81

**Expéditeur**	**Destinataire** (demandeur : nom / société / tél.)
ANSM / Direction des dispositifs médicaux thérapeutiques et des cosmétiques / Equipe produits dispositifs médicaux cardiovasculaires	Marie-Pierre BAREILLE<br>MEDES-IMPS<br>05 62 17 49 50
Dossier suivi par : Stéphanie VALLET<br>Tél : 33 (0) 1 55 87 36 57/ Fax : 33 (0) 1 55 87 37 17<br>Mel : stephanie.vallet@ansm.sante.fr	Fax **05 62 17 49 51**

**CPP** destinataire en copie	Sud-Ouest et Outre-Mer I (Toulouse 1)	**Fax**	**05.34.30.27.38**	**Code 08**

Vu le code de la santé publique et notamment ses articles L. 1123-8, R. 1123-32 et vu le dossier de demande d'autorisation d'essai clinique adressé à l'Agence nationale de sécurité du médicament et des produits de santé (ANSM) ;
Vu les compléments versés par le promoteur en date du 31 octobre 2014 et notamment le protocole de l'essai cité en objet modifié (version 5 datée du 29 octobre 2014), suite à la demande de l'ANSM ;
L'autorisation mentionnée à l'article L. 1123-8 du code de la santé publique est accordée pour l'essai clinique cité en objet. Cette autorisation est valable pour toute la durée de l'essai à compter de la date de la présente décision.
Toutefois, conformément à l'article R. 1123-33 du code de la santé publique, la présente autorisation devient caduque si la recherche n'a pas débuté dans un délai d'un an.
En outre, je vous rappelle notamment que pendant le déroulement de la recherche et pour ce qui concerne l'ANSM :

- toute modification substantielle du dossier initialement soumis doit faire l'objet d'une demande d'autorisation en vertu des articles L. 1123-9 et R. 1123-35 du code de la santé publique ;
- les effets indésirables graves inattendus ainsi que les faits nouveaux susceptibles de porter atteinte à la sécurité des personnes doivent être déclarés en vertu des articles L. 1123-10 et R. 1123-46 du code de la santé publique.

Je vous demande de transmettre toute demande d'informations complémentaires concernant ce dossier par courriel adressé à la boite : **ams-essaiscliniques**@ansm.sante.fr. Je vous précise qu'il vous est possible d'utiliser à cet effet le système de messagerie électronique sécurisée Eudralink. Lors de l'envoi de ces dossiers, je vous demande de veiller à reporter dans l'objet du message les mentions suivantes :
- pour les MS transmises à l'Ansm pour information : **MSI/ Réf ANSM du dossier**
- pour les MS soumises pour autorisation ou pour les dossiers mixtes (comportant des modifications soumises pour autorisation et d'autres pour information) : **MSA/ Réf ANSM du dossier**

La chef produits dispositifs médicaux cardio-vasculaires
Direction des dispositifs médicaux thérapeutiques,
et des cosmétiques

Si vous ne recevez pas toutes les pa[illegible]e, veuillez contacter le secrétariat de la Direction Produit Direction des dispositifs médicaux thérapeutiques et des cosmétiques au : 33 (0) 1 55 87 36 87.

Confidentialité
Cette transmission est à l'attention exclusive du(des) destinataires ci-dessus mentionné(s) et peut contenir des informations privilégiées et/ou confidentielles. Si vous n'êtes pas le destinataire voulu ou une personne mandatée pour lui remettre cette transmission, vous avez reçu ce document par erreur et toute utilisation, révélation, copie ou communication de son contenu est interdite. Si vous avez reçu cette transmission par erreur, veuillez nous en informer par téléphone immédiatement et nous retourner le message original par courrier. Merci.

Confidentiality
This transmission is intended to the addressee(s) listed above only and may contain preferential or/and confidential information. If you are not the intended recipient, you are hereby notified that you have received the document by mistake and any use, disclosure, copying or communication of the content of this transmission is prohibited. If you have received this transmission by mistake, please call us immediately and return the original message by mail. Thank you.

code : Q15CDOC004 v01

ansm
Agence nationale de sécurité du médicament et des produits de santé

Nombre de pages : 1
(incluant la page de garde)

Envoi par Télécopie

Date : 3 NOV. 2014

**Identifiants de l'essai clinique**					
Titre	EVALUATION OF THE PHYSIOLOGICAL EFFECTS OF 3 DAYS OF DRY IMMERSION USED AS A GROUND-BASED MODEL TO STUDY THE EFFECTS OF WEIGHTLESSNESS: A PILOT STUDY ON 12 HEALTHY MALE VOLUNTEERS.				
Promoteur	CNES			Réf. CPP	1-14-26
Réf. Promoteur	14-962 / DI study	N° ID RCB	2014-A00904-43	Réf. ANSM	140997B-81

**Expéditeur**	**Destinataire** (demandeur : nom / société / tél.)
ANSM / Direction des dispositifs médicaux thérapeutiques et des cosmétiques / Equipe produits dispositifs médicaux cardiovasculaires	Marie-Pierre BAREILLE<br>MEDES-IMPS<br>05 62 17 49 50
Dossier suivi par : Stéphanie VALLET<br>Tél : 33 (0) 1 55 87 36 57/ Fax : 33 (0) 1 55 87 37 17<br>Mel : stephanie.vallet@ansm.sante.fr	Fax **05 62 17 49 51**

**CPP** destinataire en copie	Sud-Ouest et Outre-Mer I (Toulouse 1)	**Fax**	**05.34.30.27.38**	**Code 08**

Madame,

L'autorisation d'essai concernant l'essai ci-dessus référencé vous a été adressée ce jour.

Toutefois, je vous rappelle la nécessité de prise de précautions concernant le stockage des bouteilles de monoxyde de carbone afin d'éviter tout accident en cas de fuite compte tenu de la toxicité de ce gaz et ainsi garantir la sécurité du personnel et des volontaires.
A ce propos, je vous invite à consulter la mise au point du 16 juillet 2012 relative aux risques et précautions d'emploi liés à l'utilisation des gaz à usage médical publiée sur le site de l'agence et accessible via le lien suivant : http://ansm.sante.fr/var/ansm_site/storage/original/application/64200b285675bf190d364fdaf0a4e4b8.pdf

Je vous prie d'agréer, Madame, l'assurance de ma considération distinguée.

La chef produits dispositifs médicaux cardio-vasculaires
Direction des dispositifs médicaux thérapeutiques,
et des cosmétiques

Nathalie MARLIAC

Si vous ne recevez pas toutes les pages de cette télécopie, veuillez contacter le secrétariat de la Direction Produit Direction des dispositifs médicaux thérapeutiques et des cosmétiques au : 33 (0) 1 55 87 36 87.

Confidentialité
Cette transmission est à l'attention exclusive du(des) destinataires ci-dessus mentionné(s) et peut contenir des informations privilégiées et/ou confidentielles. Si vous n'êtes pas le destinataire voulu ou une personne mandatée pour lui remettre cette transmission, vous avez reçu ce document par erreur et toute utilisation, révélation, copie ou communication de son contenu est interdite. Si vous avez reçu cette transmission par erreur, veuillez nous en informer par téléphone immédiatement et nous retourner le message original par courrier. Merci.

Confidentiality
This transmission is intended to the addressee(s) listed above only and may contain preferential or/and confidential information. If you are not the intended recipient, you are hereby notified that you have received the document by mistake and any use, disclosure, copying or communication of the content of this transmission is prohibited. If you have received this transmission by mistake, please call us immediately and return the original message by mail. Thank you.
