# Supplementary material for: Effects of short-term dry immersion on bone remodeling markers, insulin and adipokines
Source: PLoS One. 2017 Aug 14;12(8):e0182970. doi: 10.1371/journal.pone.0182970 (PMC5555617; doi:10.1371/journal.pone.0182970)
Supplement: S2 File — (PDF) [file pone.0182970.s002.pdf]

COMITÉ de PROTECTION des PERSONNES
SUD-OUEST ET OUTRE-MER I
Secrétariat : Mme CHÉRON - Mme VERDEILLE
AGENCE RÉGIONALE de SANTÉ MIDI PYRÉNÉES – Bureau 1028
10 chemin du raisin - 31050 TOULOUSE CEDEX 9
Tél : 05 34 30 27 55 / 05 34 30 27 56 - Fax 05 34 30 27 38 - Mail : cppsoom1-2@ars.sante.fr

Président : Denis BENAYOUN
Vice-Présidente : Catherine PIENKOWSKI
Secrétaires : Laurence NEGRE-PAGES
Jean Michel SENARD

Membres Titulaires et *Suppléants*

1er collège

* personnes qualifiées en recherche
  Catherine PIENKOWSKI
  Jean-Michel SENARD
  Philippe BOURIN
  Laurence NEGRE-PAGES
  Jeanne-Hélène di DONATO
  Eric SCHMIDT
  Emilie BERARD
  Yanina BONGARD
* médecins généralistes
  Serge ANE
  Joël LE KERNEAU
* pharmaciens hospitaliers
  Etienne CHATELUT
* infirmiers
  Christian CAZOTTES
  Frédéric DESPIAU

2ème collège

* personnes qualifiées en éthique
  Didier MERCKX
  Jean CLAVERIE
* psychologues
  Josiane PERISSE
  Béatrice HENDERSON
* travailleur social
  Julie PACOT
  Anne DALLONGEVILLE
* juristes
  Denis BENAYOUN
  Emmanuelle RIAL-SEBBAG
  Danièle CHARRAS
  Isabelle POIROT-MAZERES
* représentants d'associations et usagers de la santé
  Christophe DIVERNET
  Sylvie SFEDJ
  Marie-Ange DELORD-LEOPHONTE
  Jacques SERVILLE

MEDES - IMPS
Madame Marie-Pierre BAREILLE
BP 74404

31045 Toulouse cedex 4

Toulouse, le 26 Août 2014

Madame,

Dans sa séance du 25 Août 2014, le comité de protection des personnes (C.P.P.) Sud-Ouest et Outre-Mer I a examiné les modifications apportées au projet de recherche intitulé :

> **Evaluation of the physiological effects of 3 days of dry immersion used as a ground-based model to study the effects of weightlessness: a pilot study on 12 healthy male volunteers** – protocole 14-962 version 2 du 29 Juillet 2014

que vous lui avez soumis en qualité représentant du promoteur, le CNES, et dont l'investigateur-coordonnateur est le Dr Arnaud BECK, service de Recherche Clinique Spatiale au MEDES / IMPS

Etaient présents en qualité de titulaires : Mme PIENKOWSKI médecin, Mme BERARD médecin épidémiologiste, Mme NEGRE-PAGES épidémiologiste, Dr ANÉ médecin généraliste, Mr CHATELUT pharmacien hospitalier, Mr CAZOTTES infirmier (1er collège) ; Mr MERCKX personne compétente en matière d'éthique, Mme HENDERSON psychologue, Me BENAYOUN et Mme RIAL-SEBBAG personnes compétentes en matière juridique, Mr DIVERNET et Mr SERVILLE représentants des associations de malades et usagers de la santé (2ème collège).
Assistaient aussi à la séance, en qualité de suppléants : Mme DI DONATO scientifique, Dr SCHMIDT médecin (1er collège) ; Mr CLAVERIE personne compétente en matière d'éthique, Mme DALLONGEVILLE travailleur social, Mmes CHARRAS et POIROT-MAZERES personnes compétentes en matière juridique, (2ème collège).

Après examen de ces modifications (courrier de réponse daté et signé du 29/07/2014, la demande d'autorisation auprés de l'ANSM datée et signée du 29 juillet 2014, le document additionnel daté et signé du 26 juin 2014, la notice d'information et le formulaire de consentement - version 2 du 29/07/2014, le protocole - version 2 du 29/07/2014, le résumé - version 2 du 29/07/2014, la demande d'autorisation datée du 29/07/2014), le comité estime que :
La justification de l'étude est pertinente ; le rapport des bénéfices et des risques est acceptable.
Les objectifs de votre recherche sont bien définis et bien argumentés.
Les moyens mis en œuvre pour atteindre ces objectifs sont décrits avec suffisamment de précision et semblent bien adaptés à la solution du problème abordé.
La méthodologie est clairement décrite et adaptée aux objectifs.
La notice d'information et le formulaire de consentement sont clairement rédigés et contiennent toutes les mentions nécessaires.

En conséquence, le CPP Sud-Ouest et Outre-Mer I émet un :

AVIS FAVORABLE

à la réalisation de votre projet.
Je vous prie d'agréer, Madame, l'assurance de ma considération distinguée.

Le Président
Me D. BENAYOUN
